# Gaps in Nutrition and Exercise Counseling Preparedness among Plastic Surgery Trainees: A Nationwide Exploratory Survey Study

**DOI:** 10.1097/GOX.0000000000007807

**Published:** 2026-06-08

**Authors:** Omar Jean-Baptiste, Bo Hyun Kong, Paul Ghareeb, Yelena Burklin, Jeffrey E. Janis, Albert Losken

**Affiliations:** From the * School of Medicine, Emory University, Atlanta, GA; † Division of Plastic and Reconstructive Surgery, Emory University, Atlanta, GA; ‡ Department of Plastic and Reconstructive Surgery, College of Medicine, The Ohio State University Wexner Medical Center, Columbus, OH.

## Abstract

**Background:**

Evidence-based nutrition and physical activity influence perioperative optimization and surgical outcomes, yet formal training in these domains during plastic surgery residency remains unclear. This study surveyed US plastic surgery residents to assess their self-reported knowledge, counseling confidence, and curricular preparation.

**Methods:**

A 28-item REDCap survey was distributed to residents in the Northeastern, Southeastern, and Midwestern plastic surgery societies (September 2024–February 2025). Questions covered training level, self-rated counseling skill, 17 knowledge domains, and 4 preparation Likert scales. Categorical variables were reported as counts/percentages, and ordinal skill and curriculum preparation scores were numerically coded and reported as mean ± SD. Two-sample *t* tests compared integrated versus independent tracks and junior (postgraduate year [PGY] 1–3) versus senior (PGY ≥ 4) residents (α = 0.05).

**Results:**

Fifty-two residents responded (12% rate); 75% were in integrated programs and 73.1 % were senior (PGY 4+). Most felt comfortable with weight management counseling, but knowledge was lowest for counseling special populations (children, pregnancy, older adults). Counseling skills were largely “competent,” with 25 % (nutrition) and 31 % (physical activity) reporting “advanced” competence. Preparation scores were neutral (~3/5) and less than10% strongly endorsed curricular adequacy. Skill scores trended higher for seniors and integrated residents, but only the nutrition skill difference between senior and junior residents reached statistical significance (*P* < 0.05).

**Conclusions:**

Plastic surgery residents report moderate self-perceived preparedness and confidence in nutrition and physical activity counseling, with opportunities for enhanced curricular support. These findings highlighted the potential value of structured education to support comprehensive perioperative patient care.

**Takeaways**

**Question:** Are US plastic surgery residents adequately trained and confident in providing evidence-based nutrition and physical activity counseling to patients?

**Findings:** In a multiregional cross-sectional survey of 52 residents across three plastic surgery societies, fewer than 25% felt knowledgeable counseling children, pregnant patients, or older adults. Only 25% (nutrition) and 31% (physical activity) reported advanced counseling skill, and fewer than 10% strongly endorsed curricular adequacy. Senior residents reported significantly higher nutrition counseling skill than junior residents (*P* < 0.05).

**Meaning:** Plastic surgery residents report moderate preparedness in nutrition and physical activity counseling, suggesting a need for enhanced training in these domains during residency.

## INTRODUCTION

Evidence-based nutrition and physical activity play critical roles in perioperative optimization, wound healing, and long-term surgical outcomes across plastic surgery.^1,2^ In certain subspecialties such as burn surgery, the physiologic importance of nutrition is particularly evident, as severe burn injury induces a profound hypermetabolic and hypercatabolic state requiring aggressive nutritional management to preserve lean body mass and support recovery.^3^ As patient interest in weight management and metabolic health grows, plastic surgeons are increasingly expected to provide evidence-based guidance on nutrition and physical activity in addition to operative care.^4,5^

Plastic surgeons routinely counsel patients on perioperative risk optimization, wound healing, weight stability, and long-term outcome durability. Because plastic surgery encompasses both reconstructive and aesthetic care across diverse patient populations, preparedness in providing evidence-based nutrition and physical activity counseling represents a foundational competency extending beyond any single procedure type. These subject matters directly influence these domains, affecting complication risk, recovery, and maintenance of surgical results. Despite the clinical relevance of these factors, formal education in adequately counseling patients has historically received limited emphasis in graduate medical training, and prior studies across specialties have demonstrated variability in physician preparedness and confidence in providing such guidance.^6,7^ This disconnect may leave surgeons underprepared to provide evidence-based counseling relevant to perioperative preparation and postoperative recovery.

To better understand resident preparedness in this domain, we conducted a multi-regional, cross-sectional survey of US plastic surgery residents to assess self-reported knowledge, confidence, and counseling preparedness in evidence-based nutrition and physical activity across diverse patient populations encountered in plastic surgery practice. Our goal was 2-fold: (1) to establish the current baseline of self-reported knowledge, confidence, and counseling skill in evidence-based nutrition and physical activity and (2) to determine whether these competencies differ by residency track (integrated versus independent) and postgraduate year. Mapping these variations may help identify curricular targets and inform future efforts to strengthen surgical training in this domain.

## METHODS

### Study Design and Setting

We conducted a cross-sectional, questionnaire-based study of US plastic surgery residents. The electronic survey link was disseminated by the Northeastern, Southeastern, and Midwestern plastic surgery societies through their resident mailing lists. Where available, individual resident e-mail addresses were provided by participating societies, allowing investigators to send follow-up reminder e-mails to enhance participation. Two reminder e-mails were sent at 2-week intervals, either directly to residents when individual e-mail addresses were available or through participating societies’ mailing lists when direct contact information was not accessible. The study was approved as exempt by the Emory University institutional review board (01302023).

### Participants

All residents enrolled in Accreditation Council for Graduate Medical Education-accredited plastic surgery programs and listed on the Northeastern (132 total listed residents), Southeastern (185 total listed residents), and Midwestern society (106 listed residents) e-mail rosters were eligible. Fifty-two residents submitted complete surveys, yielding a response rate of 12% (52 of 423). Twelve partial submissions were excluded. Because the survey was offered to the entire accessible resident population through regional society mailing lists, the study was designed as an exploratory census of this population rather than a hypothesis-testing study requiring predefined sample size calculations, and therefore, no a priori power calculation was performed.

### Survey Instrument

A 28-item questionnaire was built in REDCap (v 14.0.23) with input from the Emory Center for Program Evaluation and Quality Improvement, which adapted validated prompts on nutrition and physical activity counseling. (**See appendix, Supplemental Digital Content 1**, which shows survey questions, https://links.lww.com/PRSGO/E945.) Pilot testing by three investigators required no wording changes, so pilot data were retained. The survey assessed four domains: topical knowledge (17 check-all items across nine nutrition and eight physical activity topics), counseling skill, perceived curricular preparation (four 5-point Likert items), and training characteristics (residency track and postgraduate year [PGY] level, analyzed both continuously and as junior [PGY 1–3] versus senior [PGY 4+]). Counseling skill was assessed using two 5-level self-rated items (novice, advanced beginner, competent, proficient, expert). Because no respondents selected “proficient,” the skill variable was collapsed to four ordered categories for analysis and reporting (novice, advanced beginner, competent, advanced [proficient/expert]). Because the study was exploratory and descriptive in nature, composite scores were not generated. Internal-consistency statistics were not calculated, as individual survey items were analyzed independently to assess specific domains of self-perceived preparedness.

### Data Quality, Bias Mitigation, and Confidentiality

Program evaluation and quality improvement survey specialists applied best-practice wording and pilot logic checks to minimize measurement and social desirability bias, and two scheduled reminder e-mails were issued to curb nonresponse. Survey distribution was facilitated through regional plastic surgery societies. Where societies provided individual resident e-mail addresses, investigators were able to send follow-up reminder e-mails; however, complete e-mail rosters were not available across all regions, precluding formal nonresponse bias analysis or assessment of representativeness. All responses were captured in REDCap on Emory’s 2-factor–authenticated server; access was restricted to the principal investigator and two co-investigators named on the institutional review board protocol, with audit logs recording data interactions. De-identified datasets will be archived on an encrypted institutional drive for five years in accordance with university policy.

### Statistical Analysis

Analyses were conducted in SAS 9.4 (SAS Institute, Cary, NC). Categorical data are reported as counts/percentages; ordinal skill scores and curriculum adequacy ratings were numerically coded and summarized as mean ± SD. For subgroup comparisons, counseling skill responses were treated as an ordered categorical variable and numerically coded after collapsing to 4 levels due to sparse cells (1 = novice, 2 = advanced beginner, 3 = competent, 4 = advanced [proficient/expert]). Mean Likert scores were compared with 2-sample *t* tests for (1) integrated versus independent and (2) junior (PGY 1–3) versus senior (PGY 4–fellow) residents. Normality (kernel-density plots) and equal variance (Levene test) assumptions were met, permitting treatment of Likert scales as interval data. All surveys were complete, so no imputation was required. Two-sided *P* < 0.05 defined significance; no multiplicity adjustments or sensitivity analyses were undertaken.

## RESULTS

A total of 52 plastic surgery residents completed the survey, of whom three-quarters were enrolled in integrated programs and 73.1% were senior residents (PGY 4+) (Table 1). When queried about topical knowledge, residents most frequently endorsed familiarity with weight management content (nutrition 75%; physical activity 79%) and, to a lesser extent, cardiovascular health (nutrition 48%; physical activity 64%). Fewer than 1 in 4 felt knowledgeable about counseling children, pregnant patients, or older adults in either domain, suggesting areas where respondents reported lower familiarity (Table 2). Consistent with these findings, the plurality of respondents rated their counseling skill as “competent” (nutrition 56%; physical activity 46%), with only one quarter to one-third claiming “advanced” competence and only 1 respondent in each domain reported novice-level counseling skill (Table 3).

**Table 1.** Respondent Characteristics (n = 52)

Characteristic	n (%)
Residency track	
Integrated	39 (75)
Independent	13 (25)
PGY	
1	2 (3.9)
2	3 (5.77)
3	9 (17.31)
4	8 (15.38)
5	8 (15.38)
6+/Fellow	22 (42.3)

**Table 2.** Domain‑Specific Knowledge

Knowledge Domain	n	% (of 52)
Nutrition		
Cardiovascular health	25	48.1
Weight management	39	75
Diabetes	33	63.5
Sports/athletics	31	59.7
Infants and toddlers	3	5.8
Children	7	13.5
Pregnancy	9	17.3
Older adults	9	17.3
Physical activity		
Cardiovascular health	33	63.5
Weight management	41	78.9
Diabetes	27	52.0
Sports/athletics	27	52.0
Children	8	15.4
Pregnancy	11	21.2
Older adults	12	23.1
Other (free text)	1	1.9

**Table 3.** Self‑rated Counseling Skill Levels

Skill Domain	Novice	Advanced Beginner	Competent	Advanced (Proficient/Expert)*
Evidence‑based nutrition (n = 52)	1 (1.9)	9 (17.3)	29 (55.8)	13 (25.0)
Evidence‑based physical activity (n = 52)	1 (1.9)	11 (21.2)	24 (46.2)	16 (30.8)

* Counseling skill was originally assessed using a 5-level ordinal scale (novice, advanced beginner, competent, proficient, and expert). Because no respondents selected one category, responses were collapsed into four ordered levels for analysis and reporting. Results reflect self-perceived counseling skill and do not represent objectively measured competency.

Mean curriculum adequacy scores hovered around neutral for both medical-school and residency preparation (nutrition 3.2 ± 1.0 and 3.0 ± 0.99, respectively; physical activity 3.2 ± 1.1 and 3.3 ± 0.87), and fewer than 10% “strongly agreed” that their curricula were adequate (Table 4). Mean self-rated counseling skill scores were numerically higher with seniority (nutrition 3.2 ± 0.64 versus 2.7 ± 0.83; physical activity 3.2 ± 0.72 versus 2.8 ± 0.89 for senior versus junior residents) and those in integrated programs (nutrition 3.1 ± 0.66 versus 2.9 ± 0.86; physical activity 3.1 ± 0.75 versus 2.9 ± 0.86). The difference in nutrition counseling skill between senior and junior residents reached statistical significance (*P* < 0.05), whereas other comparisons did not (Table 5).

**Table 4.** Perceived Educational Preparation (Curriculum Adequacy)

Training Source and Topic	1	2	3	4	5	Mean (± SD)
Medical-school preclinical						
Nutrition	5	20	12	14	1	3.2 (1.0)
Physical activity	7	17	10	16	2	3.2 (1.1)
Residency curriculum						
Nutrition	4	12	20	14	2	3.0 (0.99)
Physical activity	4	17	21	10	0	3.3 (0.87)

**Table 5.** Mean Self-reported Counseling Skill Scores by PGY Year and Track

Group	Nutrition Skill Score (Mean ± SD)	Physical Skill Score (Mean ± SD)
Junior residents	2.7 ± 0.83	2.8 ± 0.89
Senior residents	3.2 ± 0.64*	3.2 ± 0.72
Integrated track	3.1 ± 0.66	3.1 ± 0.75
Independent track	2.9 ± 0.86	2.9 ± 0.86

* *P* < 0.05.

## DISCUSSION

In this multiregional survey, plastic surgery residents reported moderate self-confidence and variable self-perceived preparedness in nutrition and physical activity counseling. These findings suggest that areas where residents may benefit from additional educational exposure. Approximately one quarter of respondents rated their nutrition counseling skill as “advanced,” fewer than one-third reported advanced physical activity counseling skill, and fewer than 10% believed their curricula adequately prepared them for counseling in these subjects. Survey items addressing diverse populations, including children, pregnant patients, and older adults, were intended to assess general preparedness in providing evidence-based nutrition and physical activity counseling across patient populations encountered in plastic surgery practice, rather than procedure-specific candidacy for body contouring. Seniority and integrated training were associated with numerically higher self-reported skill scores. Most subgroup comparisons did not reach statistical significance; however, senior residents reported significantly higher self-rated nutrition counseling skill compared with junior residents. These findings should be interpreted cautiously given the modest sample size and exploratory study design.

Findings from other medical specialties similarly demonstrate that physicians-in-training often feel underprepared, specifically in nutrition and exercise counseling. For example, a survey of internal medicine interns at 2 US medical residency programs found that 94% of the surveyed agreed it was a doctor’s duty to discuss nutrition with patients, yet only 14% felt that physicians are adequately trained to provide such counseling.^8^ Similarly, a nationwide survey of obstetrics and gynecology residents revealed that nearly half (48%) had no nutrition education, and less than one-third of respondents felt comfortable counseling patients on nutrition during pregnancy.^9^ Similar trends have been observed in weight management education, where trainees often report limited confidence in counseling patients on evidence-based strategies.^10^ These findings suggest that gaps in self-perceived preparedness for evidence-based nutrition and physical activity counseling are not unique to plastic surgery but represent a broader challenge in graduate medical education.

In plastic surgery specifically, perioperative counseling should include discussion of weight stability and long-term outcome durability following body contouring procedures. Prior studies have demonstrated that postoperative weight trajectory may vary by procedure type, including compensatory fat redistribution following liposuction and variable long-term weight patterns after abdominoplasty and reduction mammaplasty.^11–13^ These findings underscore the clinical relevance of nutrition and physical activity counseling to surgical outcome durability and highlight the importance of ensuring that residents are prepared to provide comprehensive perioperative guidance.

Encouragingly, educational interventions in nutrition and physical activity counseling have demonstrated measurable improvements in trainee knowledge and confidence. Targeted curricula, including online nutritional courses and structured educational workshops, have been shown to improve knowledge retention and counseling confidence in resident physicians.^14–16^ Interprofessional training models incorporating dietitians, exercise specialists, and experiential learning approaches have also improved trainee preparedness and increased comfort in providing nutrition and fitness-based counseling.^14,15^ These findings suggest that structured educational initiatives can effectively address gaps in preparedness and support improved counseling skills.

More broadly, these findings highlight the potential value of incorporating structured nutrition and physical activity education into residency training. Interprofessional curricula, modular educational programs, and experiential learning opportunities have demonstrated effectiveness in improving counseling preparedness across specialties.^17–20^ Such approaches may represent feasible strategies for strengthening resident education and supporting comprehensive perioperative patient counseling in plastic surgery. Based on these findings, several educational domains may represent opportunities for curricular enhancement. These include foundational nutrition principles relevant to perioperative optimization and wound healing, evidence-based physical activity counseling to support recovery and long-term outcomes, and practical counseling strategies to help patients maintain weight stability following surgical procedures. Structured education in these areas may help improve resident self-perceived preparedness and support comprehensive perioperative patient counseling.

### Study Limitations

This study has several important limitations. Although reminder e-mails were sent when individual contact information was available, recruitment through regional society mailing lists limited the ability to fully assess nonresponse bias or ensure representativeness of the broader US plastic surgery resident population. The response rate and modest sample size further limit generalizability and statistical power for subgroup comparisons. The cross-sectional design also prevents evaluation of how self-perceived competence evolves over time.

The survey assessed self-reported knowledge, confidence, and counseling preparedness rather than objective competency, and responses may be influenced by recall bias, social desirability bias, or differences in self-perception. Furthermore, the instrument was designed to evaluate general preparedness in evidence-based nutrition and physical activity counseling and did not assess detailed procedural physiology or pharmacologic weight management knowledge, which represent important complementary domains.

In addition, residents who elected to participate may have had greater baseline interest in lifestyle-related counseling, potentially influencing observed preparedness. The absence of reliability testing, sensitivity analyses, or correction for multiple comparisons limits the strength of secondary inferences. Finally, as an exploratory descriptive survey, the study was not powered to detect specific group differences, and subgroup comparisons should be interpreted cautiously. Given these factors, the findings should be interpreted as descriptive and hypothesis-generating rather than definitive assessments of resident competency.

## CONCLUSIONS

In this multiregional survey, plastic surgery residents reported moderate confidence and variable self-perceived preparedness in evidence-based nutrition and physical activity counseling. These findings highlight areas for future investigation and suggest potential opportunities for curriculum development to support resident education in lifestyle-related patient counseling. Further research incorporating objective knowledge assessment and broader sampling may help better characterize resident preparedness and inform future educational initiatives aimed at strengthening perioperative counseling skills in plastic surgery training. Strengthening education in evidence-based nutrition and physical activity counseling represents an opportunity to further support comprehensive perioperative patient care within plastic surgery training. Future multi-institutional studies with larger and more representative samples will be essential to confirm these findings and guide evidence-based curricular development in plastic surgery training.

## FUNDING

This study was supported in part by the National Center for Advancing Translational Sciences of the National Institutes of Health under award number UL1TR002378 and by TL1TR002382.

## DISCLOSURES

Dr. Losken serves on the Advisory Boards for Bimini and Novus. These relationships are unrelated to the present work. Dr. Janis receives royalties from Thieme and Springer Publishing. The other authors have no financial interest to declare in relation to the content of this article.

## Supplementary Material


