# Supplementary material for: Gaps in Nutrition and Exercise Counseling Preparedness among Plastic Surgery Trainees: A Nationwide Exploratory Survey Study
Source: Plast Reconstr Surg Glob Open. 2026 Jun 8;14(6):e7807. doi: 10.1097/GOX.0000000000007807 (PMC13246045; doi:10.1097/GOX.0000000000007807)
Supplement: Supplementary file 1 [file gox-14-e7807-s001.pdf]

Survey Questions:

Thank you for participating in this brief survey. The following questions will help to establish plastic surgery residents' current level of knowledge and acquired skills related to evidence-based nutrition, physical activity, and weight management.

Your responses are anonymous. The survey should take **less than 5 minutes** to complete. We greatly appreciate your participation!

If you agree to complete this survey, please click the arrow to start.

DEMOGRAPHICS

1. Please indicate your residency track:

    a) Integrated
    b) Independent

2. What is your current year in residency?

    a) PGY-1
    b) PGY-2
    c) PGY-3
    d) PGY-4
    e) PGY-5
    f) PGY-6
    g) PGY-7
    h) PGY-8
    i) PGY-9 or above

ATTITUDES

3. Please select the phrase that best describes your current skill level in counseling patients on evidence-based nutrition:

    a) Novice – I have no knowledge of evidence-based nutrition and would not feel confident discussing it with patients
    b) Advanced beginner – I have limited knowledge of evidence-based nutrition and would not feel confident discussing it with patients
    c) Competent – I have basic knowledge of evidence-based nutrition and can hold a conversation about it with patients
    d) Proficient - I have advanced knowledge of evidence-based nutrition and can help patients problem-solve in this area
    e) Expert - I have extensive training in evidence-based nutrition and can provide nutrition counseling to patients

4. Please select the phrase that best describes your current skill level in counseling patients on evidence-based physical activity:

a) Novice – I have no knowledge of evidence-based physical activity and would not feel confident discussing it with patients
b) Advanced beginner – I have limited knowledge of evidence-based physical activity and would not feel confident discussing it with patients
c) Competent – I have basic knowledge of evidence-based physical activity and can hold a conversation about it with patients
d) Proficient - I have advanced knowledge of evidence-based physical activity and can help patients problem-solve in this area
e) Expert - I have extensive training in evidence-based physical activity, and can provide physical activity counseling to patients

5. My medical school's preclinical curriculum adequately prepared me to address patient questions about evidence-based nutrition.
a) Strongly agree
b) Agree
c) Neither agree nor disagree
d) Disagree
e) Strongly disagree

6. My medical school's preclinical curriculum adequately prepared me to address patient questions about evidence-based physical activity.
a) Strongly agree
b) Agree
c) Neither agree not disagree
d) Disagree
e) Strongly disagree

7. My Plastic Surgery residency curriculum adequately prepared me to address patient questions about evidence-based nutrition.
a) Strongly agree
b) Agree
c) Neither agree nor disagree
d) Disagree
e) Strongly disagree

8. My Plastic Surgery residency curriculum adequately prepared me to address patient questions about evidence-based physical activity.
a) Strongly agree
b) Agree
c) Neither agree not disagree
d) Disagree
e) Strongly disagree

9. Which areas, if any, do you have knowledge about? *Select all that apply*
- Nutrition for cardiovascular health
- Nutrition for weight management
- Nutrition for diabetes
- Nutrition for sports/athletics
- Nutrition for infants and toddlers
- Nutrition for children
- Nutrition during pregnancy
- Nutrition for older adults

- Physical activity for cardiovascular health
- Physical activity for weight management
- Physical activity for diabetes
- Physical activity for sports/athletics
- Physical activity for children
- Physical activity during pregnancy
- Physical activity for older adults
- Other (please specify*)

We thank you for your time spent taking this survey. Your response has been recorded.
